# Update on recommendations for the diagnosis and treatment of SARS-CoV-2 infection in children

**DOI:** 10.1007/s10096-020-03973-x

**Published:** 2020-08-06

**Authors:** Hongjun Miao, Han Li, Yinying Yao, Mingfu Wu, Chao Lu, Jun Wang, Man Tian, Ying Li, Peiliang Luo, Jianhui Gu, Bin Yuan, Shouchuan Wang, Xia Zhao, Weihua Gan, Deyu Zhao

**Affiliations:** 1 Department of Emergency, Children’s Hospital of Nanjing Medical University, Nanjing, China; 2 Department of Pediatrics, The Affiliated Hospital of Yangzhou University, Yangzhou, China; 3 Department of Pediatrics, The First Affiliated Hospital of Nanjing Medical University, Nanjing, China; 4 Department of Pediatrics, The Affiliated Hospital of Xuzhou Medical University, Xuzhou, China; 5 Department of Respiratory, Children’s Hospital of Nanjing Medical University, Nanjing, China; 6 Department of Emergency, Children’s Hospital Affiliated to Suzhou University, Suzhou, China; 7 Department of Pediatrics, The First People’s Hospital of Lianyungang, Lianyungang, China; 8 Department of Pediatrics, The Affiliated Hospital of Nantong University, Nantong, China; 9 Department of Pediatrics, The Affiliated Hospital of Nanjing University of Traditional Chinese Medical, Nanjing,, China; 10 Department of Pediatrics, The First Clinical Medical College of Nanjing University of Traditional Chinese Medical, Nanjing, China; 11 Department of Pediatrics, The Second Affiliated Hospital of Nanjing Medical University, Nanjing, China

**Keywords:** New coronavirus, Child, Diagnosis and treatment, Recommendation, Update

## Abstract

Since the outbreak of novel coronavirus infection pneumonia in Wuhan City, China, in late 2019, such cases have been gradually reported in other parts of China and abroad. Children have become susceptible to severe acute respiratory syndrome coronavirus 2 (SARS-CoV-2) because of their immature immune function. As the outbreak has progressed, more cases of novel coronavirus infection/pneumonia in children have been reported. Compared with adults, the impact of SARS-CoV-2 infection in children is less severe, with a lower incidence and susceptibility in children, which results in fewer children being tested, thereby underestimating the actual number of infections. Therefore, strengthening the diagnosis of the disease is particularly important for children, and early and clear diagnosis can determine treatment strategies and reduce the harm caused by the disease to children. According to *the Novel Coronavirus Infection Pneumonia Diagnosis and Treatment Standards* (*trial version 7*) issued by National Health Committee and the latest diagnosis and treatment strategies for novel coronavirus infection pneumonia in children, this review summarizes current strategies on diagnosis and treatment of SARS-CoV-2 infection in children.

## Epidemiological characteristics of SARS-CoV-2 infection

### Infection sources

Patients are the main source of infection. From the incubation period to the recovery period before the respiratory virus nucleic acid is negative for two consecutive times, they are infectious and highly infectious. Asymptomatic infections are common in pediatrics. The incubation period of SARS-CoV-2 infections ranges from 1 to 14 days, mostly ranging from 3 to 7 days. Currently, the longest incubation period was reported as 24 days [1, 2].

### Transmission route

The main transmission ways of the virus are respiratory droplets and contact transmission, and the possibility of transmission by aerosol exists under conditions of prolonged exposure to high concentrations of aerosol in a relatively closed environment. The novel coronavirus can be isolated from feces and urine; therefore, attention should be paid to the aerosol or contact transmission caused by feces and urine to pollute the environment. Mother-to-child transmission and other transmission routes need to be further identified [3].

### Susceptible population and epidemic characteristics

There are general susceptibilities in all groups, and children are no exception. Current reported data of infected populations revealed that the ages of those infected with the disease ranged from 36 h to 96 years, with no significant gender difference. There are less pediatric cases, and less severe, although some severe pediatric cases have been reported. In this outbreak, there are obvious features of clustering, and the number of imported cases or those transmitted by second-generation transmission is rapidly increasing and is highly contagious. Close family contact is the main mode of infection in children [4]. Deaths in children have been reported to date [5].

## Etiology and pathological mechanism

After SARS⁃CoV⁃2 comes into contact with the human respiratory tract, the spike protein of the virus binds to the surface receptor of sensitive cells, angiotensin-converting enzyme 2 (ACE2), which mediates viral entry into type II alveolar epithelial cells to facilitate virus proliferation and spread. ACE2 is highly expressed on alveolar cells in Asian males [6]. Recently, it has been reported that the S protein of SARS-CoV-2 binds to ACE2 with a higher affinity than SARS coronavirus, which might contribute to the rapid spread of COVID-19 [7]. Currently, it is unclear whether children’s low susceptibility to SARS-CoV-2 is related to the development and function of ACE2. SARS-CoV-2 can destroy lymphocytes. Therefore, the absolute number of lymphocytes in peripheral blood in adults is significantly or progressively reduced, and the subsets of CD4 ^+^ and CD8 ^+^ T cell subsets are all decreased. While the white blood cell count and absolute number of lymphocytes in children are mostly normal [8]. SARS-CoV-2 infection is also associated with an inflammatory cytokine storm, which is characterized by elevated levels of in various inflammatory factors. Serum fibrinous exudate and hyaline membrane formation are observed in the alveolar cavity; exudative cells consist mainly of monocytes and macrophages, and multinucleated giant cells are easily observed. Part of the epithelium of the bronchial mucosa in the lungs is shed, and the formation of mucus and mucus plugs in the cavity is visible. The lungs show varying degrees of consolidation, with a few manifestations of excessive alveolar inflation, alveolar septum rupture, or cyst cavity formation [3].

## Clinical manifestations

At the onset of the disease, infected children mainly present with fever and cough, which might be accompanied by systemic symptoms, such as fatigue, myalgia, headache, dizziness, nausea, vomiting, abdominal pain, diarrhea, nasal congestion, runny nose, sneezing, and sore throat. Moreover, their thermal pattern is irregular. They could present with high, medium, or low fever, even no fever. Symptoms of some children and neonates are not typical, presenting with gastrointestinal symptoms, such as vomiting and diarrhea or only with mental weakness and shortness of breath. The current case analysis shows that in children, the duration of fever is mostly 1–2 days, with a maximum of 8 days. In the early phase of the disease, the total number of white blood cells in peripheral blood is normal or decreased, with a decreased lymphocyte count [9, 10]. Dyspnea, cyanosis, and other symptoms can occur as the condition aggravates, usually after 1 week of the disease. The condition of some children might progress rapidly and could develop into respiratory failure that cannot be corrected by conventional oxygen (nasal catheter, mask) within 1–3 days, which indicates that the child is critically ill. In these severe cases, septic shock, irreversible metabolic acidosis, and bleeding and coagulation dysfunction may occur [11]. At the same time, children with other (i.e., comorbid) diseases (such as congenital heart, lung and airway diseases, malnutrition, and tumors, etc.) are vulnerable to infection with SARS-CoV-2; more specifically, “prone to severe illness.” Older children might have similar forms as adults. Compared with adults, children with COVID-19 were mild symptoms, faster recovery, shorter detoxification time, and good prognosis [12, 13]. Neonates, especially preterm infants, who are more likely to present with insidious and non-specific symptoms, need closer observation. According to the clinical characteristics of existing cases of infected children, they can be divided into the following clinical types:

1. Asymptomatic infection

The patient’s SARS-CoV-2 nucleic acid test was positive, but there are no clinical symptoms.

2. Mild type

The main manifestation in these patients is acute upper respiratory infection. Pharyngeal hyperemia is observed on physical examination with no positive sign in the lungs.

3. Ordinary type

Symptoms include fever, cough, pharyngeal pain, nasal congestion, fatigue, headache, and myalgia. Some patients show signs of pneumonia on chest imaging; however, but no dyspnea and other hypoxia symptoms are observed, and their general condition is good [14].

4. Severe type [3]

The disease progresses quickly and meets any of the following conditions:

1. Significantly increased respiration rate (RR): RR ≥ 60/min (< 2 months), RR ≥ 50/min (2~12 months old), RR ≥ 40/min (1~5 years old), RR ≥ 30/min (> 5 years old), after ruling out the effects of fever and crying [15].
2. Hypoxia: having assistant respiration chosen from groaning, nasal flaring and three depression sign, cyanosis, and pulse blood oxygen saturation (SpO_2_) ≤ 92% (< 90% in premature infants).
3. Consciousness disorders: apathy, somnolence, coma, and convulsions.
4. Food refusal or feeding difficulty and dehydration.
5. Other manifestations: such as bleeding and coagulation disorders (prolonged prothrombin time and elevated levels of D-dimer), myocardial damage (increased level of myocardial enzymes and troponin, electrocardiogram ST-T changes, cardiomegaly, and cardiac insufficiency in severe cases), gastrointestinal dysfunction, raised level of liver enzyme, and rhabdomyolysis.
6. Critical cases [16]

Disease progresses rapidly to organ failure with any of the following conditions:

1. Respiratory failure that requires mechanical ventilation: patients present with acute respiratory distress syndrome (ARDS) featured by refractory hypoxemia.
2. Septic shock: in addition to severe pulmonary infection, SARS-COV-2 can also cause damage and dysfunction of other organs. When dysfunction of the extrapulmonary system, such as circulation, blood, and digestive system, occurs, the possibility of sepsis and septic shock should be considered and the mortality rate increases significantly.
3. Accompanied by other organ failure that requires intensive care unit (ICU) monitoring and treatment.

## Severe and critical clinical warning indicators [3]

1. Increased breathing rate
2. Poor mental response, lethargy
3. Progressive elevation of lactic acid levels
4. Chest imaging findings indicating bilateral or multi-lobe infiltration, pleural effusion, or rapid progression of conditions during a very short period
5. Infants younger than 3 months or those with existing underlying diseases (e.g., congenital heart disease, bronchopulmonary dysplasia, respiratory tract malformation, abnormal hemoglobin, and severe malnutrition), and immune deficiency or low immunity (long-term use of immunosuppressants)

## Auxiliary examinations

### Routine laboratory examinations [3, 11, 14]

1. Routine blood test: the total number of white blood cells is normal or decreased, accompanied by lymphocyte reduction; progressive lymphocytopenia might be observed in severe cases. The normal values of children at different ages are different from the adult standards, which should be taken into consideration.
2. C-reactive protein (CRP): normal or increased.
3. Erythrocyte sedimentation rate (ESR): elevated in most children.
4. Procalcitonin (PCT): normal in most cases. A level of PCT > 0.5 ng/mL indicates coinfection with bacteria.
5. Others: Elevation of liver enzymes, lactate dehydrogenase, muscle enzymes, and myoglobin; an increased level of D-dimer might be seen in severe cases.
6. Elevated inflammatory cytokines.

### Etiological detection

Nucleic acid testing is the main method of laboratory diagnosis. SARS-COV-2 nucleic acid can be detected from throat swabs (nasopharyngeal swabs are recommended for children), sputum, lower respiratory tract secretions, stool, urine, tears, or blood samples, by real-time quantitative polymerase chain reaction (RT-PCR); or by viral gene sequencing [3]. Nasopharyngeal swab tests are more common in China. The stool sample is as specific as the nasopharyngeal swab [13].

#### Other methods

SARS-CoV-2 particles can be isolated from human respiratory epithelial cells through virus culture, but this experiment cannot be carried out in general laboratories [11].

### Imaging features [11, 17, 18]

#### Chest X-ray examination

In the early stage of COVID-19, chest images show that the texture of the two lungs increased and became rough, followed by the appearance of small patchy shadows and interstitial changes, especially in the lung periphery. Severe cases can further develop to bilateral multiple ground-glass opacity and pulmonary consolidation, with infrequent pleural effusion.

#### Chest CT scan

Pulmonary lesions are shown more clearly by CT, including ground-glass opacity and segmental consolidation in bilateral lungs, especially in the lung periphery. In children with a severe infection, multiple lobar lesions may be present in both lungs. Compared with those in adults, most pediatric patients are mild type whose chest CT characteristics of COVID-19 were atypical, with more localized round glass opacity (GGO) extent, lower GGO attenuation, and relatively rare interlobular septal thickening. In addition, the imaging is required in asymptomatic children with SARS-CoV-2 infection.

## Diagnosis [3, 15, 19]

### Suspected cases

It is necessary to perform a comprehensive analysis based on epidemiological history and clinical manifestations.

#### Epidemiological history

1. Within 14 days before disease onset, children with a travel or residence history in China or a country or region with a serious epidemic where the infection cases was reported.
2. Within 14 days before disease onset, children with a history of contacting novel coronavirus infected cases (children with positive test for viral nucleic acid).
3. Within 14 days before disease onset, children with a history of contacting patients with fever or respiratory symptoms who have a travel or residence history in China or a country or region with a serious epidemic where the infection cases was reported.
4. Children who are related to a cluster outbreak: two or more cases with fever and/or respiratory symptoms occurred within 2 weeks in small areas such as home, office, and school classes.

#### Clinical features

1. Fever and/or respiratory symptoms
2. With imaging findings of pneumonia caused by SARS-CoV-2
3. In the early phase of the disease, white blood cell counts are normal or decreased, mostly with decreased lymphocyte count and normal or increased CRP

Patients should be suspected of SARS-CoV-2 infection if they meet any one of the criteria in the epidemiological history and any two of the criteria in clinical manifestations. Newborns born to pregnant women with SARS-CoV-2 nucleic acid positive are designated as suspected cases.

Note: Due to the presence of asymptomatic infection that meets the clinical characteristics of (3), no special pneumonia etiological examination, ineffective treatment with oseltamivir and other drugs, and chest X-ray examination consistent with the characteristics of COVID-19, suspected cases should be considered for appropriate etiological examination, even if the child has no clear epidemiological history.

### Confirmed case

**Fig. 1:**
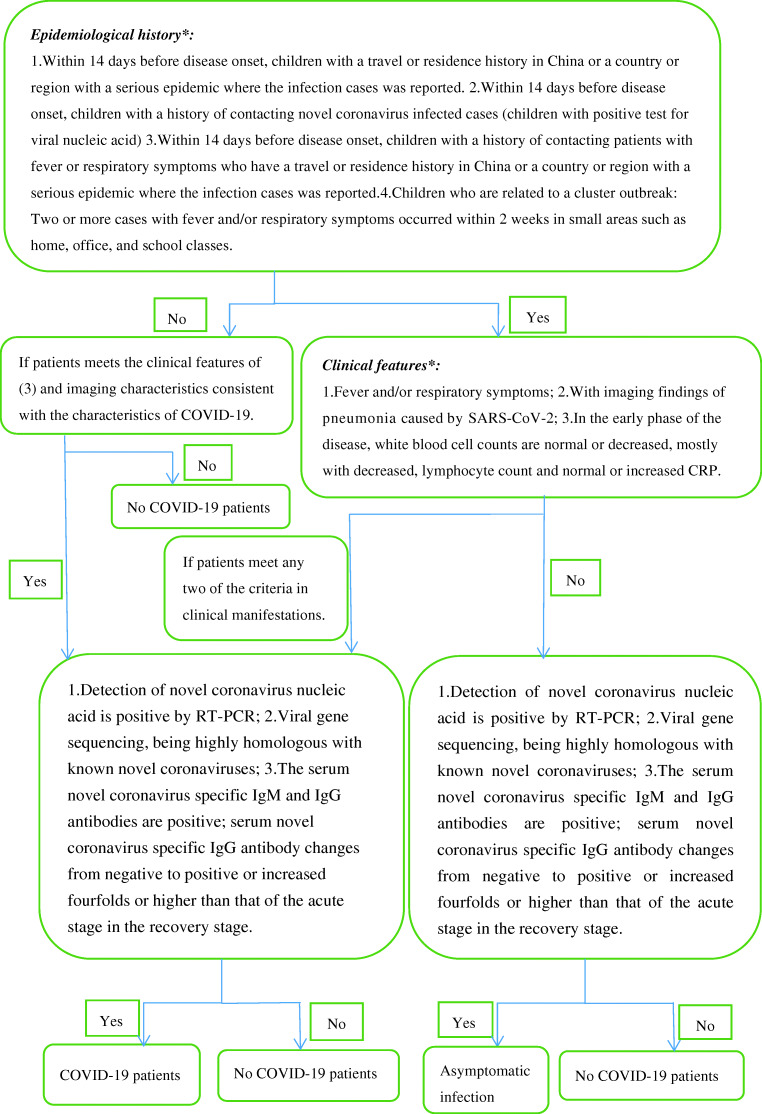
Diagnostic algorithm of COVID-19. Note: (1) Newborns born to pregnant women with SARS-CoV-2 nucleic acid positive are designated as suspected cases. (2) Asymptomatic infection who meets the clinical characteristics of (3), no special pneumonia etiological examination, ineffective treatment with oseltamivir and other drugs, and chest X-ray examination consistent with the characteristics of COVID-19 should be considered as suspected cases, even if the child has no clear epidemiological history

Suspected cases become confirmed cases after one of the following etiological or serological evidence:

1. Detection of novel coronavirus nucleic acid is positive by RT-PCR. (Note: rectal swab testing by RT-PCR in children may be more useful than nasopharyngeal swab testing by RT-PCR in determining the effectiveness of treatment and determining the timing of quarantine termination [13].)
2. Viral gene sequencing, being highly homologous with known novel coronaviruses
3. The serum novel coronavirus-specific IgM and IgG antibodies are positive; serum novel coronavirus-specific IgG antibody changes from negative to positive or increased fourfold or higher than that of the acute stage in the recovery stage (Fig. 1)

### Differential diagnosis

SARS-CoV-2 infection should be distinguished from adenovirus, influenza virus, respiratory syncytial virus, and mycoplasma pneumonia. In addition, attention should be paid to the identification of human avian influenza virus, parainfluenza virus, human metapneumovirus, human bocavirus, and other viral pneumonia, bacterial pneumonia, fungal pneumonia, legionella, and tuberculosis [19]. It should also be distinguished from non-infectious diseases such as vasculitis, dermatomyositis, and organizing pneumonia [3]. SARS-CoV-2 infection can also be mixed with other bacteria or viruses or have a double infection. Attention should be paid to differential diagnosis and treatment [11].

1. Adenovirus pneumonia: The disease is more common in 6 months to 2 years old children, with the appearance of continued high fever, drowsiness and dispirited, pale complexion, severe cough, wheezing, and difficulty breathing. Signs in the lungs appear late, and moist crackles are heard 4 to 5 days after fever onset. Signs of consolidation of the lungs are found after lesion fusion. Blood tests show that the numbers of white blood cells are mostly reduced or normal. Imaging findings show patchy fuzzy shadows or large leaf consolidation [20].
2. Influenza virus pneumonia: There may be persistent high fever, and coughing is obvious. Other symptoms included fatigue, diarrhea, and chest pain. There are small wet moist crackles in the lungs. Routine blood examination shows that the white blood cell count is mostly normal or slightly increased, although some white blood cells are reduced. X-ray manifestations of the chest show irregular flocculent shadows on the lungs on both sides of the hilum, which are not widely distributed [21].
3. Respiratory syncytial virus pneumonia: Children with this infection are usually younger, less than 2 years old, especially below 6 months. Clinical features are prone to wheezing, with or without fever, and wheezing and dyspnea in severe cases. Wheezing sounds and fine moist crackles can be heard upon auscultation. Chest radiographs can show hyperventilation and small patchy shadows. The course of the disease is self-limiting [22].
4. Mycoplasma pneumonia: The disease may occur in any season. Patients are predominantly school-age children; however, the number of cases among younger children is increasing. Infection usually starts with high fever and a cough gradually develops. Lung signs are relatively few. Chest images may reveal manifestations including reticular shadows and small patchy or large consolidation. Routine blood examination shows an increased or normal white blood cell count and CRP is slightly elevated [11].

## Case discovery and reporting (isolation measures)

### Case detection, reporting, and isolation [3, 13]

During the daily diagnosis and treatment in medical institutions at various levels, upon finding suspected cases that meet the conditions of the disease, an in-hospital specialist or an attending physician consultation should be conducted immediately. If the suspected cases are still considered, the appropriate organization should report directly to the network within 2 h and collect samples to detect of novel coronavirus nucleic acid as soon as possible. At the same time, the suspected case is immediately transferred to a single person single room in the designated hospital for isolation and treatment under the premise of ensuring the safety of the transfer. The medical staff then implements secondary or higher protection and should pay attention to the strict disinfection disposal of patient excreta and secretions.

When two consecutive rectal swab novel coronavirus nucleic acid tests (the sampling interval is at least 24 h) and the specific antibodies IgM and IgG detection of novel coronavirus 7 days after the onset of the disease are still negative in the suspected case, the diagnosis of suspected case can be excluded.

### Medical observation [5, 23, 24]

1. Close contacts or suspected exposures should be subject to medical observation at home or in a single isolated room. The medical observation period is 14 days after the last ineffective protective contact or suspected exposure with a confirmed patient.
2. Newborns delivered by confirmed mothers in China: The child should be separated immediately from the mother for isolation and observation after birth (secondary protection level). There continue to be conflicting data as to the role of breastfeeding on transmitting neonatal-maternal infection. United Nations International Children’s Emergency Fund (UNICEF) recommends continuing with breastfeeding, while applying necessary precautions to prevent transmission of infection. In contrast, the Chinese Working Group for the Prevention and Control of Neonatal SARS-CoV-2 Infection recommends milk formula for every child of a mother who has been infected.
3. Healthy newborns at term: After 1 week of isolation observation in hospital, if the child has a negative test for viral nucleic acid twice, normal feeding, and good general condition, they can be isolated for medical observation at home. If the child develops symptoms at home, they should return to the hospital for examination immediately.
4. Premature infants or pathological neonates with asphyxia and other diseases should be isolated in a single room and receive appropriate treatment.
5. Monitoring of SARS-CoV-2 virus nucleic acid: Within 24 h, 5–7 days, and 14 days after birth, respiratory tract secretions are taken for detection of viral nucleic acid. Any positive test for viral nucleic acid should be reported immediately, and the health and disease conditions should be re-evaluated to decide whether the infant can stay at home or be hospitalized for isolation treatment. The treatment method is the same as that for other children. Patients who test negative for virus nucleic acid for the third time can be released from quarantine.

### Treatment

These patients with asymptomatic infection, mild infection (e.g., fever, cough, and/or myalgias without dyspnea, hypoxia, and tachypnea), and having no underlying condition should only be isolated in the outpatient setting without special treatment. Other patients should be treated with drugs or organ support [25].

### Medical isolation

The four principles of early recognition, early isolation, early diagnosis, and early treatment should be emphasized. Medical isolation is supposed to be carried out once the suspected cases are identified. Suspected and confirmed cases should be admitted to designated hospitals. Suspected case should be isolated in a single room, while confirmed cases can be arranged in the same room [11].

### General treatment

The general therapeutic strategies include bed rest; monitoring vital signs; strengthening supportive treatment; ensuring sufficient calories; maintaining homeostasis such as water, electrolytes, and acid-base balance; and maintaining an unobstructed respiratory tract. Older children should be supported using psychotherapy.

### Oxygen therapy

Patients with hypoxemia (SpO_2_ < 95%) should receive oxygen therapy, including nasal catheter and mask oxygen, and whether respiratory distress and/or hypoxemia are alleviated should be assessed promptly. Tracheal Intubation and non-invasive or invasive mechanical ventilation should be undertaken for patients with severe disease instantly [8, 26, 27].

### Antiviral treatment

There are currently no effective antiviral drugs to treat COVID-19 for children (Table 1).

**Table 1:** Antiviral drugs of COVID-19

Antiviral drugs	Dosage	Indications	Side effects
Interferon α	200,000 to 400,000 IU/kg in 2 mL sterile water, with nebulization two times per day for 5–7 days	Covid-19, chronic hepatitis B and C patients, etc.	Fever, headache, nausea, vomiting, lack of concentration, and even pessimism
Lopinavir/litonavir	Weight 7–15 kg, 12 mg/3 mg/kg; weight 15–40 kg, 400 mg/100 mg as adult each time, twice a day for 1–2 weeks	COVID-19 and HIV patients	Diarrhea, nausea and vomiting
Ribavirin	10 mg/kg/time, via intravenous infusion, 2 to 3 times daily	COVID-19 patients, viral pneumonia, and bronchitis caused by respiratory syncytial virus	Teratogenic and hemolytic anemia
Remdesivir	–	COVID-19 patients	Skin rash, diarrhea, and renal dysfunction
Favipiravir	–	COVID-19 patients	Skeletal muscle atrophy and myocardial necrosis
Chloroquin phosphate/Hydroxychloroquine	3–5 mg/kg/day (max dose 400 mg) hydroxychloroquine sulfate iv, twice daily for 5 days	COVID-19 and malaria patients	QT interval prolongation, ventricular arrhythmias and other cardiac toxicity

#### Interferon-α

Interferon can inhibit virus replication in a variety of ways. In the early stage of infection, interferon can reduce the viral load, which can help to alleviate symptoms and shorten the course of the disease. However, coronavirus infections cannot be targeted. Interferon-α atomization can be tried, such as 200,000 to 400,000 IU/kg or 2 to 4 μg/kg in 2 mL sterile water, with nebulization two times per day for 5–7 days [26]. However, the drug increases the risk of fever, headache, nausea, vomiting, lack of concentration, and even pessimism.

#### Lopinavir/litonavir

Lopinavir, which is a protease inhibitor of human immunodeficiency virus 1(HIV-1), is commonly used in combination with ritonavir, which increases the half-life of lopinavir by inhibiting cytochrome P450. Ex vivo experiments indicated that lopinavir/ritonavir could inhibit coronavirus replication to a certain extent [28]. Attempts have been made to use this drug to treat adult patients with pneumonia caused by SARS-CoV-2 [29]. A recently published systematic review revealed that the effect of this drug combination is mainly manifested in its early application, which can reduce patient mortality and glucocorticoid dosage. However, if the early treatment window is missed, late application has no significant effect. The use of lopinavir/litonavir in children should be undertaken with caution, and children with severe disease can be considered as appropriate. It is recommended that the usage and dosage of lopinavir /ritonavir in children can refer to the guidelines for anti-HIV therapy. The recommended doses: weight 7–15 kg, 12 mg/3 mg/kg; weight 15–40 kg, 10 mg/2.5 mg/kg; weight > 40 kg, 400 mg/100 mg as adult each time, twice a day for 1–2 weeks. Please pay attention to the possible side effects such as diarrhea and nausea and vomiting during use. Children with liver disease should contraindicate this drug [11, 27].

#### Ribavirin

Ribavirin, as a broad-spectrum potent antiviral drug, belongs to the group of drugs acting on the synthesis of nucleosides, which have inhibitory effects on many DNA and RNA viruses [30]. However, in vitro experiments showed that high doses of ribavirin have inhibitory effects on coronaviruses. It is recommended to use it in combination with interferon-α or lopinavir/ritonavir, at 10 mg/kg/time, via intravenous infusion, 2 to 3 times daily [14]. A critically ill child can be given antiviral therapy with ribavirin injection empirically, at 15 mg/kg each time, twice a day, and the course of treatment should not exceed 5 days [31]. At the same time, in view of the side effects of ribavarin, including the teratogenic and hemolytic anemia, it should be used with caution in pregnant women and children.

#### Remdesivir

Remdesivir, a new generation of ribavirin agents, is a nucleoside analog that can integrate into the nascent viral RNA strand and lead to premature termination of viral replication [32, 33]. However, unlike ribavirin, it has strong antiviral activity in vitro against human infection with coronaviruses and various bat-derived coronaviruses [32]. In addition, its efficacy is better than that of lopinavir/litonavir combined with interferon-beta [34]. There are currently reports of its effective use in related treatment; however, further clinical validation is needed [33]. However, the patients who take the medicine have the risk of skin rash, diarrhea, and renal dysfunction. In severe cases, septic shock, acute renal injury, multiple organ dysfunction syndrome, and other side effects may also occur.

#### Favipiravir

Favipiravir (FPV) is an RNA-dependent RNA polymerase inhibitor. It has now been shown to be useful against SARS-CoV-2 in initial clinical trials. Some studies have found that compared to Lopinavir/Ritonavir, the FPV-treated patient demonstrated much better therapeutic response especially with regard to faster viral clearance and improvement rate in chest imaging [35]. At present, there is no specific dosing recommendation for FPV in pediatric COVID-19 patients [25]. But we should be alert to the possibility of skeletal muscle atrophy and myocardial necrosis.

#### Chloroquin phosphate

The use of chloroquine phosphate for antiviral therapy has been added to the *Diagnosis and Treatment of Pneumonia Caused by 2019- nCoV* (*trial version 6*) [36]. Chloroquine phosphate has been confirmed to be resistant to a variety of viruses in vitro. This drug has multiple activities, one of which is to alkalize the phagolysosome, which hampers the low-pH-dependent steps of viral replication [37]. Studies have found that chloroquine, at an EC_50_ of 1.1 μM, is effective in preventing replication of this novel coronavirus [38]. But there are in vitro studies that show hydroxychloroquine appears to be more potent [39]. Some experts from Iran suggested to use 3–5 mg/kg/day (max dose 400 mg) hydroxychloroquine sulfate iv, in pediatric patients, twice daily for 5 days [25]. However, the drug has been independently shown to increase the risk for QT interval prolongation, ventricular arrhythmias, and other cardiac toxicity [40].

#### Other drugs

##### Antibacterial agents

The blind or irrational use of antimicrobials, especially in combination with broad-spectrum antimicrobials, should be avoided. Close attention should be paid to changes in the condition of children with bacterial or fungal coinfection; samples should be collected actively for pathogen analysis and the timely or rational use of antibiotics or anti-fungal drugs [41, 42].

##### Arbidol, oseltamivir, and other anti-influenza drugs

Arbidol is administrated for adults infected with SARS-CoV-2; however, its efficacy and safety remain unclear. Oseltamivir and other anti-influenza agents can be applied in patients coinfected with other influenza viruses [43, 44].

##### Glucocorticoids

When using glucocorticoids, it is necessary to strictly monitor the indications, combined with the severity of the disease, and comprehensively consider the following indicators: Fever over 38.5 °C for 3 days, CRP ≥ 30 mg/L, serum ferritin ≥ 1000 μg/kg, the rapid progressing of imaging findings, significant hypoxia, patients manifesting the symptoms of ARDS, and obvious wheezing. Methylprednisolone can be used for short periods (3–5 days). The recommended dose of methylprednisolone should not exceed 1–2 mg/kg/day [8, 27, 44].

##### Gamma globulin for injection

For severe and critical cases, of the use gamma globulin can be considered; however, its efficacy remains unclear at present. The recommended dose is 1.0 g/kg/day for 2 days, or 400 mg/kg/day for 5 days [11, 44].

##### Convalescent plasma

Blood transfusion therapy (anti-serum) of convalescent patients can be considered for severe and critically ill patients [3]. However, the collection of convalescent plasma must be conducted at the appropriate time to ensure that it has a high neutralizing antibody titer, so as to significantly improve the survival rate of patients [10].

##### Immunotherapy

For adults with extensive lesions of both lungs, severely ill patients, and for those with laboratory tests showing elevated interleukin (IL)-6 levels, tocilizumab treatment can be tried. When using this drug, attention should be paid to allergic reactions, and people with active infection such as tuberculosis are contraindicated [3]. The efficacy and safety of this drug in children remains to be determined; therefore, it should be used caution.

##### Vaccines

Vaccines have the effect of preventing infection, reducing disease severity, and viral shedding. The main antigens for vaccine development are the structural spike glycoprotein S or its receptor-binding domain (RBD). Several vaccines against SARS-CoV-2 are in development, including the classical inactivated and attenuated vaccines, the protein subunit and virus-like particle vaccines (VLP), viral vector-based vaccines, as well as the newer DNA- and RNA-based vaccines. So far, some vaccines has entered into clinical trials, such as mRNA-1273 vaccine, Ad5-nCoV, etc. [35, 45].

## Treatment of patients with severe and critical disease

### Basic principles

On the basis of symptomatic treatment, these measures, including treatment of underlying diseases, active comprehensive treatment to correct hypoxemia, the provision of effective organ function protection, and preventing disease complications need to be implemented.

### Respiratory support

1. Severely ill children should use a nasal cannula or simple continuous positive airway pressure (CPAP) to inhale oxygen. CPAP generally has a pressure of 5–10 cmH_2_O. When refractory hypoxemia or ARDS (according to the latest PARDS diagnostic criteria in 2015 [46]) occurs, it is critical that timely blood gas analysis and assessment of the condition are performed. The patient in critical condition should be transferred to the pediatric intensive care unit (PICU) in time to adjust the oxygen therapy plan, including high-flow nasal cannula oxygen therapy or non-invasive mechanical ventilation. If hypoxia symptoms cannot be corrected within 1 to 2 h, invasive mechanical ventilation should be performed [36, 44]. For children with respiratory failure and/or circulatory failure that cannot be improved after the above treatment, the use of extracorporeal membrane oxygenation (ECMO) can be considered [3, 11, 36].
2. Invasive mechanical ventilation strategy: PaO_2_ is maintained at 55 ~ 80 mmHg or SpO_2_ at 88 to 95%. The lung protective ventilation strategy is adopted, in which small tidal volume ventilation for 3 to 8 mL/kg is provided and the plateau pressure is 28 cmH_2_O on the inhale. The level of plateau pressure can increase to 29~32 cmH_2_O for children with reduced chest wall compliance. The best positive end-expiratory pressure (PEEP) can be selected according to the PEEP-FiO_2_ table method. Patients with moderate to severe ARDS can use a higher PEEP (> 12 cmH_2_O) in the early stage. For children with severe ARDS, it is recommended to perform lung recruitment, adjust the positive airway pressure to 30~45 cmH_2_O for 30~40 s, and set the inspiratory pressure at 40 cmH_2_O for 40 s. It is recommended that prone position ventilation is provided for more than 12 h every day [3, 19]. But it is important to note that the hemodynamics are stable.
3. Indications for ECMO: No improvement after mechanical ventilation and other treatment methods, progressive deterioration of the P/F or oxygen index (OI), PaO_2_/FiO_2_ < 60 mmHg or OI > 35 for more than 6 h, or severe respiratory acidosis (pH < 7.15), circulation function that cannot be improved, basal blood pressure maintained by a large number of vasoactive drugs, or continuously elevated blood lactic acid levels, and other conditions [11]. When the underlying disease is controlled and the cardiopulmonary function shows signs of recovery, withdrawal of the machine test can be started [3].
4. Sedative muscle relaxation and artificial hibernation therapy: Patients undergoing mechanical ventilation or receiving ECMO need to be sedated using analgesia. For patients who are very resistant during the establishment of an artificial airway, short-term application of low-dose muscle relaxants is recommended. Hibernation therapy is recommended for patients with severe disease and a low oxygenation index. Artificial hibernation therapy can reduce the body’s metabolism and oxygen consumption, while simultaneously dilated the pulmonary blood vessels to significantly improve oxygenation. It is recommended to supply a continuous intravenous bolus, and closely monitor the patient’s blood pressure. To prevent the occurrence and exacerbation of lung infections, we should try to avoid prolonged excessive sedation, and withdraw muscle relaxants as soon as possible when conditions permit. It is recommended to closely monitor the depth of sedation [31].

### Circulatory support

Consciousness, skin condition, pulse, capillary filling time, urine volume, and blood lactic acid levels should be closely observed to identify shock early. If the heart function is normal, the septic shock procedure can be followed. If ARDS is present, strict fluid management should be performed to ensure tissue perfusion to maintain a negative liquid balance, and to actively treat capillary leakage and maintain heart and kidney function. Hemodynamics need to be closely monitored during anti-shock therapy [3, 44].

### Other organ function support

Organ function support is used to closely monitor the organ functions of children, including the nervous system, digestive system, urinary system, blood system, coagulation function, water-electrolyte acid-base balance, endocrine internal environment, and others, and then provide corresponding treatment.

### Blood purification

Blood purification can clear inflammatory factors and block the “cytokine storm,” thereby relieving the damage caused by inflammatory responses to the body. It can be used to treat mid to early cytokine storms in severe and critically ill patients [3]. When multiple organ failure (especially acute kidney injury) is involved, or when capacity overload and life-threatening water, electrolyte, or acid-base imbalances are considered, continuous blood purification treatment should be considered [11]. If combined with liver failure, plasma exchange is feasible [47].

## Psychotherapy [26, 44]

We must integrate families, schools, and psychologists to provide children with comprehensive psychological and life support.

### Children who have been isolated

During this period, children are prone to fear, anxiety, depression, and insomnia [48]. The main intervention measures are:

1. Provide basic living and treatment security for the isolation of sick children.
2. Provide psychological support outside the medical care team for the isolated children, encourage children more, and give children companionship outside medical operation hours.
3. Assist in communicating with parents and transmitting information, such as video calls, and timely transmission of classmates’ support information.
4. Explain the importance and necessity of isolation and encourage children to build confidence that they will recover their health as soon as possible.

### Healthy children whose parents or guardians are passively isolated due to illness

Children have a number of worries and anxieties about their parents’ health, their parents leaving, loneliness, and being abandoned. Anxiety, depression, and even post-traumatic stress disorder can occur in severe cases. The main interventions are:

1. According to the children’s age-related cognition level, different notification methods are used to let them know why they are separated from their parents, such that children realize that their parents are sick and need treatment, rather than abandoning them.
2. Provide suitable living places, strengthen communication with other relatives, and set up relatively stable care team members as far as possible.
3. Assist in communicating with parents who are physically fit in isolation to ease anxiety caused by separation.
4. Continue to follow a regular routine.
5. Consult the psychiatric department if necessary.

## Remove isolation and discharge standards [3, 36, 44, 49]

The body temperature of the confirmed cases returned to normal for more than 3 days. The respiratory symptoms improved significantly, and the pulmonary imaging showed obvious absorption of inflammation. The nucleic acid test for respiratory pathogens was negative at two consecutive time points (with a sampling interval of at least 24 h). If these conditions are met, patients can discharge from isolation or transferred to the appropriate department for treatment of other diseases according to their condition.

## Precautionary measures [11, 26, 44]

On the one hand, it is necessary to control the source of infection, and children infected by novel coronavirus should be isolated at home or admitted to designated hospitals under the guidance of medical staff. On the other hand, it is necessary to block the transmission routes, prevent respiratory tract or contact transmission, reduce exposure to infection, and avoid going to public places. In addition, it is necessary to protect vulnerable groups, eat a balanced diet, exercise adequately, and rest regularly.

## Data Availability

Data sharing not applicable to this article as no datasets were generated or analyzed during the current study.
